# *Clostridium septicum* growth from a total knee arthroplasty associated with intestinal malignancy: a case report

**DOI:** 10.1186/1471-2334-12-235

**Published:** 2012-10-02

**Authors:** Demetri M Economedes, Jerome Santoro, Carl A Deirmengian

**Affiliations:** 1 Department of Orthopaedic Surgery, Philadelphia College of Osteopathic Medicine, 4190 City Avenue, Philadelphia 19131, Pa, USA; 2 Department of Medicine, Lankenau Medical Center, 100 Lancaster Avenue, Wynnewood, 19096, Pa, USA; 3 Department of Orthopaedic Surgery, Lankenau Medical Center, 100 Lancaster Avenue, Suite 456, Wynnewood, 19096, Pa, USA

**Keywords:** *Clostridium septicum*, Total Knee Arthroplasty, Infection, Malignancy

## Abstract

**Background:**

Previous reports of infection with *Clostridium septicum* have identified an unexpected association with concurrent malignancy. The reported rate of associated malignancy has been found to be as high as 81 percent. The purpose of this case report was to present a case of a total knee arthroplasty infected with C. *septicum* and the subsequent finding of an occult colonic malignancy.

**Case presentation:**

A 74 year-old man underwent uneventful bilateral total knee arthroplasties. Two weeks post-operatively, he presented with acute swelling, redness and pain of the left knee. Aspiration of the knee was sent for cell count and culture. The cell count demonstrated 39,000 white blood cells per cubic millimeter with 71% of white blood cells identified as neutrophils. Synovial fluid cultures identified the presence of C. *septicum*, Enterobacter and coagulase negative Staphylococcus. After urgent irrigation and debridement and polyethylene exchange of the affected knee, the patient was placed on intravenous Penicillin G for a period of six weeks. Two weeks into his course of antibiotics, the patient developed hematochezia and was found to have an obstructive colonic malignancy. The patient underwent hemi-colectomy and has since made a complete recovery of both his malignancy and total knee arthroplasty infection.

**Conclusion:**

Recognition of the association between C. *septicum* and malignancy is especially important considering the large predicted increase in total joint arthroplasty procedures over the coming decades. In addition to the standard treatment for infection after total joint arthroplasty, identification of *Clostridium septicum* should initiate a search for associated occult malignancy.

## Background

Infections involving clostridial organisms have been well described in the orthopaedic literature, primarily in the setting of post-traumatic wounds. Although *Clostridium perfringens* is the usual culprit in these lesions, there have been approximately 169 reports of patients with non-traumatic gas gangrene caused by *Clostridium septicum*. Previous reports of infection with C. *septicum* have identified an unexpected association with concurrent malignancy. In a review of the literature from 1945–1987, Kornbluth et al. reported a rate of associated malignancy of 81 percent [1].

We report the case of a 74-year-old man with an acute postoperative infection after total knee arthroplasty (TKA). C. *septicum* was one of the organisms identified, and the patient was subsequently found to have a colonic malignancy. To our knowledge this is the second report in the literature of C. *septicum* identified from a synovial fluid culture after total knee arthroplasty as Burnell et al., published the first report in 2011 [2]. The patient was informed that the data concerning the case would be submitted for publication, and he consented.

## Case presentation

A 74-year-old man initially presented to our office with severe bilateral knee pain and radiographs revealing significant arthritic changes. The patient’s past medical history at that time included only asthma and benign prostatic hypertrophy for which he was receiving regular medical treatment. Standard preoperative medical evaluation and laboratory testing revealed no further abnormalities, and he subsequently underwent bilateral TKA. During the immediate post-operative period, the patient was diagnosed with non-insulin dependent diabetes, but otherwise progressed normally and was discharged to an in-patient rehabilitation unit without complication.

Two weeks post-operatively, the patient developed acute swelling, redness and pain of the left knee. Aspiration of the knee was sent for cell count and culture. The cell count demonstrated 39,000 white blood cells per cubic millimeter with 71% of white blood cells identified as neutrophils. Based on the local signs of infection and the significantly elevated synovial fluid white blood cell count, the patient was taken urgently to the operating room for an irrigation, synovectomy, debridement and polyethylene exchange. Cultures were sent from the operating room, and the patient was started on Vancomycin while cultures were pending. Synovial fluid cultures identified the presence of C. *septicum*, Enterobacter and coagulase negative Staphylococcus and blood cultures also revealed growth of C. *septicum*. *C. septicum* was found to be susceptible to Clindamycin and Penicillin. Given the presence of a clostridial species, consideration was given to proceeding with an urgent aggressive debridement with removal of implants, however the patient’s knee had appeared quite well since the debridement, and there were no signs of a worsening clinical picture. The intravenous antibiotic was changed to Penicillin G and was continued over a standard six week course. A future colonoscopy was scheduled by the department of infectious diseases, considering the presence of *Clostridium septicum,* and the patient was discharged back to an inpatient rehabilitation unit with an uncomplicated hospitalization.

Two weeks after irrigation and debridement, the patient had an episode of hematochezia and was subsequently hospitalized. During this hospitalization, the patient was diagnosed with an obstructive colon cancer, located at the hepatic flexure and distal transverse colon. The patient underwent a right transverse and partial left colectomy with nodal biopsy. Final pathology demonstrated both lesions to be adenocarcinomas, which were moderately differentiated and invasive, without nodal disease. There were no distant metastatic lesions seen on CT scan of the chest, abdomen and pelvis. Given this patient’s histologic grade, lack of metastatic disease and gross pathologic findings, he was not deemed a candidate for post-operative chemotherapy. The patient’s post-operative course was complicated by the finding of bilateral popliteal deep venous thrombi, which were treated with a retrievable inferior vena cava filter and warfarin for six months. At six months follow-up from hemi-colectomy, blood work and CT scan of the abdomen and pelvis demonstrated no evidence of recurrence or metastatic disease.

Nine months after the irrigation and debridement, the patient presented with a reinfected TKA, requiring a two-stage revision procedure for persistent chronic infection. *C. septicum* was not identified in the synovial fluid or tissue cultures at the time of antibiotic spacer block implantation. The patient underwent reimplantation of a revision knee arthroplasty, has now been off of antibiotics and asymptomatic for seventeen months.

## Conclusion

To our knowledge this is the second reported case of a total joint arthroplasty infected by *Clostridium septicum*. This case underscores the importance of searching for malignancy in a patient with a C. *septicum* infection, as this was the first sign of malignancy in this patient. Given the dramatically increasing rates of total joint arthroplasty and infected total joint arthroplasties, awareness of the association between C. *septicum* and malignancy is critical to the early treatment and survival of a patient with intestinal malignancy.

Clostridial infection has long been associated with post-traumatic and surgical wound complications, as this gram positive, anaerobic, spore former is usually found in the soil and gastrointestinal tract. C. *perfrigens* is the most common species, as it is involved in 80-90% of all Clostridial infections. C. *septicum* on the other hand, is a species that is isolated at a rate of only 4-20% of Clostridial infections [3]. Although only found in a fraction of patients infected with Clostridial species, Kornbluth et al., found a survival rate of only 35% of those who were infected with C. *septicum* regardless of the presence of an occult malignancy versus a clinically evident malignancy. Therefore, it is essential to follow a systematic and aggressive approach to the care of these patients [1].

C. *septicum* infection has been documented to have an 85% concurrence with malignancy, as initially described by Alpern and Dowell in 1969 and confirmed twenty years later by Kornbluth et. al [1,4]. While the association of malignancy and infection has been proven, it is not exactly clear how the organism gains entry to the bloodstream. C. *septicum* was initially thought to be part of the normal flora of the gastrointestinal tract, however this has been recently questioned as multiple studies have found no definitive existence of C. *septicum* in stool cultures [5,6]. It has been established that Clostridia species will only grow in areas of necrosis [7]. Therefore, it has been theorized that its initial growth occurs within necrotic tissue of the occult bowel malignancy and is then disseminated throughout the body. Clostridial proliferation is not only limited to areas of tumor necrosis, but is also common in areas of necrosis secondary to pseudomembranous colitis, agranulocytic colitis and ischemic colitis [8]. Interestingly, C. *septicum* infection has not been established in iatrogenic forms of immunosuppression.

The first reported case of septic arthritis caused by C. *septicum* was in a 5-year-old boy in 1983 with a traumatic etiology. The authors did acknowledge the strong association of occult malignancy with this infection, however, it was not reported whether the patient underwent a malignancy work-up or was ever diagnosed in follow-up [9].

Macy et al. and Fallon et al. were first to document an atraumatic septic arthritis caused by C. *septicum* in a 71-year-old male in 1986. After irrigation and debridement the patient was placed on antibiotics and a work-up for malignancy was conducted. An occult adenocarcinoma of the right colon was discovered, for which the patient underwent successful hemicolectomy and recovered without complication [10,11].

Since 1986, there have only been two reported cases of nontraumatic septic arthritis caused by C. *septicum*[2,12]. In the first case, an 85-year-old female presenting with increasing knee pain was diagnosed with septic arthritis. Her case was complicated by anemia secondary to melena for which she received multiple transfusions. She died on hospital day twenty-six due to congestive heart failure. Post-mortem autopsy revealed caecal malignancy with local lymph node involvement [12]. In the second case, Burnell et al. reported the first case of C. septicum infecting a total knee arthroplasty in 2011. They described an 87-year-old female with acute hematogenous infection of a well functioning total knee arthroplasty done 11 years previously. The patient underwent an initial irrigation and debridement of her left total knee arthroplasty with retention of all components and was placed on intravenous cefazolin and metronidazole. At the discretion of the Infectious Disease physician she underwent CT scan of the abdomen and subsequent endoscopy. She was found to have a cecal mass of moderate to poorly differentiated adenocarcinoma. The patient went onto fail the initial irrigation and debridement of the total knee arthroplasty at 4 weeks post-op which necessitated two-stage exchange of the prosthesis. The patient expired after placement of the antibiotic spacer and awaiting second stage reimplantation secondary to metastatic disease involving the liver and adrenal glands [2].

Recognition of the association between C. *septicum* and malignancy is especially important considering the large predicted increase in total joint arthroplasty procedures over the coming decades [13]. In addition to the standard treatment for infection after total joint arthroplasty, identification of *Clostridium septicum* should initiate a search for associated occult malignancy. An abdominal CT-scan during the initial hospitalization in this case may have obviated the need for subsequent readmission and provided an earlier diagnosis of an intestinal mass. Fortunately, in this current case report, the malignancy was discovered early enough to provide what appears to be a cure. Additionally, this case underscores the importance of isolating and identifying all organisms and sub-species within a poly-microbial infection, as the isolation of C. *septicum* will generate the additional concern of an underlying occult malignancy.

## Competing interest

DE and JS have no competing interests. CD is the majority stockholder of CD Diagnostics however, this was not a competing interest in relation to this manuscript.

## Authors’ contributions

DE carried out the data collection, literature review and drafting of the manuscript. JS contributed to the drafting of the manuscript and aided in the literature review. CD conceived the study, participated in the data collection and the drafting of the manuscript. All authors read and approved the final manuscript.

## Pre-publication history

The pre-publication history for this paper can be accessed here:

http://www.biomedcentral.com/1471-2334/12/235/prepub
